# Phylogenomic and biogeographic reconstruction of the *Trichinella* complex

**DOI:** 10.1038/ncomms10513

**Published:** 2016-02-01

**Authors:** Pasi K. Korhonen, Edoardo Pozio, Giuseppe La Rosa, Bill C. H. Chang, Anson V. Koehler, Eric P. Hoberg, Peter R. Boag, Patrick Tan, Aaron R. Jex, Andreas Hofmann, Paul W. Sternberg, Neil D. Young, Robin B. Gasser

**Affiliations:** 1 Faculty of Veterinary and Agricultural Sciences, The University of Melbourne, Melbourne, Victoria 3010, Australia; 2 Istituto Superiore di Sanità, Viale Regina Elena 299, 00161 Rome, Italy; 3 Yourgene Bioscience, Shu-Lin District, New Taipei City 23863, Taiwan; 4 United States National Parasite Collection, US Department of Agriculture, Agricultural Research Service, Beltsville, Maryland 20705, USA; 5 Department of Biochemistry and Molecular Biology, Monash University, Melbourne, Victoria 3800, Australia; 6 Genome Institute of Singapore, 60 Biopolis Street, Singapore 138672, Republic of Singapore; 7 Cancer and Stem Cell Biology, Duke-NUS Graduate Medical School, Singapore 138672, Republic of Singapore; 8 Structural Chemistry Program, Eskitis Institute, Griffith University, Brisbane, Queensland 4111, Australia; 9 Division of Biology, Howard Hughes Medical Institute, California Institute of Technology, Pasadena, California 91125, USA

## Abstract

Trichinellosis is a globally important food-borne parasitic disease of humans caused by roundworms of the *Trichinella* complex. Extensive biological diversity is reflected in substantial ecological and genetic variability within and among *Trichinella* taxa, and major controversy surrounds the systematics of this complex. Here we report the sequencing and assembly of 16 draft genomes representing all 12 recognized *Trichinella* species and genotypes, define protein-coding gene sets and assess genetic differences among these taxa. Using thousands of shared single-copy orthologous gene sequences, we fully reconstruct, for the first time, a phylogeny and biogeography for the *Trichinella* complex, and show that encapsulated and non-encapsulated *Trichinella* taxa diverged from their most recent common ancestor ∼21 million years ago (mya), with taxon diversifications commencing ∼10−7 mya.

## Abstract (web summary)

Trichinellosis is a globally important food-borne disease caused by roundworms of the *Trichinella* complex. Here the authors present genomic sequences representing all 12 recognized *Trichinella* species and genotypes, and reconstruct their phylogeny and biogeography.

## Graphical abstract

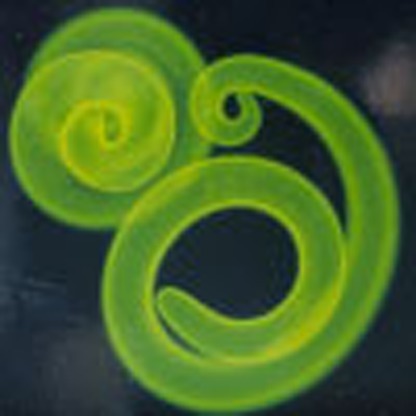

Parasitic diseases cause substantial morbidity and mortality in billions of animals and humans worldwide, and also major losses to the global food production annually. Parasitic roundworms (nematodes) of the genus *Trichinella* cause disease (trichinellosis) in humans, which can lead to serious morbidity and mortality^1^,^2^. Although trichinellosis is endemic in many regions of the world, the predominant impact of human disease relates principally to acute outbreaks following consumption of infected, raw meat products^2^, with examples in Argentina, China, Laos, Papua New Guinea, Romania and Vietnam^3^,^4^,^5^,^6^,^7^,^8^,^9^.

*Trichinella* is a complex of at least 12 species and genotypes, with a broad geographic range, including Europe, Africa, Asia, the Americas and Australasia^10^,^11^. Although only morphologically distinguishable groups (that is, encapsulated and non-encapsulated clades) of *Trichinella* are recognized, based on the appearance of larvae in muscle cells in infected hosts, molecular studies have defined nine species and three genotypes that display extensive biological diversity^10^,^11^,^12^,^13^. Currently, based on genetic data, the encapsulated clade (infecting only mammals) includes *T. spiralis*, T1; *T. nativa*, T2; *T. britovi*, T3; *T. murrelli*, T5; *T. nelsoni*, T7; *T. patagoniensis*, T12; and *Trichinella* genotypes T6, T8 and T9; and the non-encapsulated clade includes *T. pseudospiralis*, T4 (infecting mammals and birds); *T. papuae*, T10; and *T. zimbabwensis*, T11 (infecting mammals and reptiles). These taxa quite often represent localized populations, and exhibit varying degrees of intraspecific genetic variability (*T. pseudospiralis*), dispersal ability and host usage^12^.

The biological diversity of *Trichinella* is of major evolutionary significance and reflects substantial genetic diversity, divergent ecology and host–parasite affiliations^11^,^14^,^15^. Despite some advances, there have been significant knowledge gaps in the biogeography and phylogeny of the *Trichinella* complex, as most previous studies represent analyses of small-scale molecular data sets, limiting interpretation and conclusions^1^,^2^,^3^,^4^,^5^,^6^,^7^. Progress in genomic, transcriptomic and bioinformatic technologies^16^,^17^,^18^ now provides unique opportunities to rapidly overcome such limitations and to enable research on *Trichinella* and trichinellosis. Although the nuclear genome of *T. spiralis*^19^ and the mitochondrial genomes of most *Trichinella* taxa have been characterized^20^,^21^, there have been no global, nuclear genomic or transcriptomic data sets for the 11 other recognized taxa of *Trichinella*. In the present study, we sequenced and assembled draft genomes and transcriptomes for all currently recognized *Trichinella* species and genotypes and defined protein-coding gene sets. Using these data sets, we then reconstructed the phylogeny and biogeography of *Trichinella*, as a basis to address some pertinent and outstanding questions regarding the evolution and biology of *Trichinella*.

## Results

### Genomes and transcriptomes

We sequenced and assembled 16 draft genomes and 15 transcriptomes from all 12 currently recognized *Trichinella* taxa, including five distinct geographic isolates of *T. pseudospiralis* (Tables 1 and 2 and Supplementary Tables 1–4). The draft assemblies of these genomes (available publicly; see ‘Accession codes' section) ranged from 46.1 to 51.5 Mb (mean: 49.0 Mb), with average scaffold N50s of 106–294 kb (mean: 196 kb) and GC contents of 32.5–33.7% (33.2%; Tables 1 and 2). For all assemblies, 96.4% (95.6–97.2%) of all 248 core essential genes were identified (Tables 1 and 2), indicating that the gene sets represent substantial proportions of individual genomes. The repeat contents of individual draft genomes were estimated at 6.7–21.8% (mean: 17.7%) of their total nucleotide compositions (Supplementary Tables 5 and 6). On average, the assemblies contain 2.4% (range: 1.1–3.7%) retrotransposons, 2.9% (0.7–5.5%) DNA transposons, 7.7% (0.04–10.6%) unclassified dispersed elements and 4.8% (4.1–5.8%) simple repeats (Supplementary Tables 5 and 6). The repeat content of the genome of genotype T9 (6.7%) is exceptionally low compared with those of all other *Trichinella* taxa (mean: 17.7%). A comparison of the present draft genome for *T. spiralis* (ISS3) with that published previously for same species (ISS195)^19^ revealed essentially the same percentage of core essential genes (96%), and repeat and GC contents, but a size difference (14 Mb), likely to relate to differing sequencing and assembly methodologies or a genetic distinctiveness between the two geographic isolates.

### Protein-encoding gene sets

From the 16 genomes representing all *Trichinella* taxa, we predicted 11,006–16,067 (mean: 13,912) protein-encoding genes that were 2,071–3,169 bp in length (mean: 2,632; with introns), exons that were 170–223 bp in length (mean: 203) and introns that were 218–284 bp in length (mean: 259), with 5.7–6.9 exons per gene using complementary, *de novo* and homology-based approaches (Tables 1 and 2). These genes and introns are shorter than those of *Trichuris suis* (mean: 3,812 and 510 bp), *Haemonchus contortus* (6,167 and 832 bp), *Ascaris suum* (mean: 6,636 and 1,081 bp) and *Toxocara canis* (8,416 and 1,133 bp), but are similar to those of *Brugia malayi* (3,106 and 311 bp) and *Caenorhabditis elegans* (3,680 and 320 bp)^22^,^23^,^24^,^25^,^26^,^27^,^28^. In total, 7,659–11,931 (mean: 9,697; 69.9%) of the protein-encoding genes had homologous sequences (BLASTp *E* value: 10^−5^) in the NCBI nr protein database (7,655–11,930; mean 9,694; 69.7%), UniProtKB/SwissProt database (5,143–5,912; mean: 5,603; 40.3%) and WormBase (4,891–5,415; mean: 5,205; 37.4%). Genes with domain and motif matches in InterProScan (*n*=5,289–6,372; mean: 5,847; 42.0%) contained 4,626–5,362 (5,035; 36.2%) hits in Pfam, 5,018–5,879 (5,489; 39.5%) in PANTHER, 929–1,037 (992; 7.1%) in PRINTS and 234–266 (252; 1.8%) in PIRSF databases (Supplementary Tables 7 and 8). In total, 7,691–12,000 (mean: 9,730) orthologous genes (*E* value≤10^−5^) were associated with biological (KEGG) pathways. The secretomes of individual *Trichinella* taxa were predicted to comprise 314–414 (mean: 363) excretory/secretory (ES) proteins, 238 of which are encoded by single-copy orthologous genes (SCOs; Supplementary Data 1) and include cysteine, metallo- and serine proteases, peptidase inhibitors and orphan (uncharacterized) proteins identified previously^29^,^30^,^31^,^32^.

### Phylogeny

**Figure 1:**
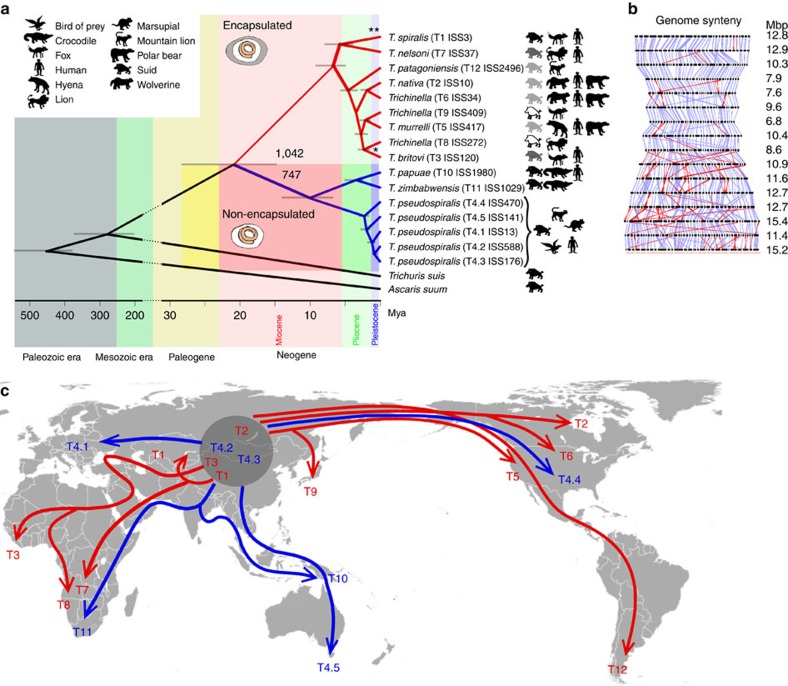
The evolution and biogeography of *Trichinella* taxa. (**a**) The phylogeny of all 12 currently recognized taxa of *Trichinella* based on analyses of amino-acid sequence data from shared SCOs (*n*=1,284) employing Bayesian infererence, ML and MP methods, with *Trichuris suis* and *A. suum* as outgroups; 1,042 and 747 are the numbers of orthologous gene groups, which are unique to encapsulated (red) and non-encapsulated (blue) *Trichinella* taxa, respectively. The topology of the trees constructed using each of these methods was the same; all nodes have absolute statistical support (1.00 or 100%), except for one node (*) in the ML analysis, where it was 99%. The grey bars on the nodes represent 95% confidence intervals for the estimate of species branching time. *T. spiralis* (ISS195)^19^ shares the same phylogenetic position (**) as *T. spiralis* (ISS3). Host animals: suids (*Sus scrofa*) represent both the sylvatic and domestic porcine hosts (left); the reproductive potential of a particular *Trichinella* taxon in *S. scrofa*^12^ is indicated by the colour scale: white: not assessed; light grey: low; dark grey: medium; black: high. Other animals (right) represent examples of carnivorous sylvatic hosts in different geographic regions, including fox, lion, mountain lion, marsupial, crocodile and bird of prey, and the accidental human host. (**b**) Representation of genome-wide synteny among *Trichinella* taxa (same order as listed on the right in **a**). Genomic scaffolds (black) sharing at least 10 SCOs between *Trichinella* taxa are displayed. A purple line indicates a single SCO and a red line an inverted SCO. The numbers on the right indicate the genomic length in megabases (Mb). (**c**) Biogeography of *Trichinella* taxa proposed on the basis of known global (climate, extinctions and/or tectonic) events and diversification times (mya) for *Trichinella* taxa, estimated using a molecular clock approach. Encapsulated taxa: *T. spiralis*, T1; *T. nativa*, T2; *T. britovi*, T3; *T. murrelli*, T5; *T. nelsoni*, T7; *T. patagoniensis*, T12; and *Trichinella* genotypes T6, T8 and T9. Non-encapsulated taxa (infecting mammals, reptiles and/or birds): *T. pseudospiralis*, T4; *T. papuae*, T10; *T. zimbabwensis*, T11 (ref. 12). Geographic distributions of *Trichinella* taxa were reported by Pozio and Zarlenga^11^. The embedded public domain world map image ( https://commons.wikimedia.org/wiki/File:BlankMap-World6.svg) has been modified using the programs World map tool v.1.16 ( http://law.nagoya-u.ac.jp/en/appendix/software/worldmap) and GIMP v.2.8 ( https://www.gimp.org).

We reconstructed the phylogeny of all 12 currently recognized *Trichinella* taxa (*cf.* Tables 1 and 2). First, we conducted an analysis of a complete protein sequence data set representing all 1,284 SCOs shared by all taxa, including five distinct geographic isolates of *T. pseudospiralis*, and two outgroup species (that is, *Trichuris suis* and *A. suum*) using Bayesian inference, maximum likelihood and maximum parsimony to establish the phylogenetic relationships of all encapsulated and non-encapsulated *Trichinella* taxa. Here all three trees constructed had the same topology, with strong support values (1.0; 87–100%) at all nodes, irrespective of the algorithm used (Fig. 1a). Second, employing the same algorithms, we independently assessed the relationships of all 16 *Trichinella* gene sets (without an outgroup) using protein sequences encoded by 2,855 SCOs shared by all taxa. Again, all three resultant trees had the same topology, consistently achieving very strong nodal support (1.0; 94–100%), with the exception of the pair *T. spiralis*+*T. nelsoni* in the maximum parsimony analysis (support: 64%). Third, to address this discrepancy, we studied separately the relationships of all encapsulated taxa using protein sequences encoded by 4,090 SCOs, and achieved strong support at all nodes for the three algorithms (1.0; 99–100%). Then, to assess the root relationship of sister species *T. spiralis*+*T. nelsoni* to non-encapsulated clades, we employed the same approach to resolve the relationships of three encapsulated (*T. spiralis*, *T. nelsoni* and *T. patagoniensis*) and one non-encapsulated taxon (*T. papuae*) using protein sequences encoded by 4,300 SCOs, and achieved the same outcome (that is, absolute support at all nodes). The final consensus tree constructed (Fig. 1a) represents the results from the first and second analyses (for all three algorithms). As expected, the dendrogram constructed from syntenic correlation values (Supplementary Data 2) was consistent in topology with this consensus tree and supported the encapsulated and non-encapsulated clades (Fig. 1a).

For the encapsulated taxa, the consensus tree shows that a lineage with *T. spiralis*+*T. nelsoni* is the sister to other taxa, which hierarchically include *T. patagoniensis*, and pairs of sister species (*T. nativa*+T6, *T. murrelli*+T9 and *T. britovi*+T8; Fig. 1a). These relationships differ from those inferred in previous studies using few ribosomal or mitochondrial DNA sequences^10^,^14^,^15^, although some previously recognized sister species associations are concordant, and *T. spiralis*+*T. nelsoni* and *T. patagoniensis* are relatively basal. At the time of initial phylogenetic assessment of *Trichinella*^14^, sequence data were not available for all five *T. pseudospiralis* isolates (T4.1–T4.5) or *T. patagoniensis*. Here, for non-encapsulated taxa, the pair *T. papuae*+*T. zimbabwensis* is basal to all representatives of *T. pseudospiralis* investigated (that is, T4.1–T4.5); T4.4 from North America is basal to T4.5 from Tasmania and to all three isolates from Eurasia (T4.1–T4.3; Fig. 1a). Using the amino-acid sequences encoded by shared SCOs (*n*=2,855), tree topology and nodal support values were similar to those achieved using whole-mitochondrial data sets^21^, although these values were consistently high compared with previous analyses using small gene sets^14^,^15^. Here, for non-encapsulated taxa, the pair *T. papuae*+*T. zimbabwensis* is basal to all representatives of *T. pseudospiralis* investigated (that is, T4.1–T4.5); T4.4 from North America is basal to T4.5 from Tasmania and to all three isolates from Eurasia (T4.1–T4.3; Fig. 1a). Therefore, we have now been able to establish, for the first time, the phylogenetic relationships of all currently recognized *Trichinella* taxa, including five *T. pseudospiralis* representatives (that is, T4.1–T4.5) and the more recently discovered *T. patagoniensis*.

### Divergence and biogeography

Divergence time analysis based on the nematode diversification estimate of 532–382 million years ago (mya)^33^ (1,000 SCOs) implied that *Trichinella* and *Trichuris suis* had a most recent common ancestor (MRCA) ∼281 mya (95% credible interval: 384–204), and that the encapsulated and non-encapsulated *Trichinella* taxa shared an MRCA ∼21 (28–15) mya (Fig. 1a), coinciding with the transition from Oligocene to Miocene^34^. Relative to a deep age for the *Trichinella* lineage, origin of a specific adaptation for encapsulation, associated with the radiation of nine taxa, occurred late in the evolutionary history of these nematodes. Subsequent diversification leading to extant species or species groups within the encapsulated and non-encapsulated clades is temporally circumscribed in the upper and uppermost Miocene during the Tortonian and Messinian periods^35^,^36^, commencing ∼7 (9–5) and 10 (14–7) mya (Fig. 1a) and continuing into the Pliocene and Pleistocene^34^. Despite major methodological differences, our estimates are very consistent with those of Zarlenga *et al.*^14^ in postulating a geographic distribution for the MRCA of all *Trichinella* taxa in Eurasia. Further concordance is seen in estimates for initial divergence of respective clades for encapsulated and non-encapsulated forms during the mid-Miocene, coincidental with perturbations in temperate ecosystems before diversification of extant species (Fig. 1a,c); this scenario might relate to an early Miocene glaciation^37^, with low sea levels^38^ allowing a regional interchange of faunas linking Africa, Eurasia and North America^39^. The diversification of non-encapsulated *T. pseudospiralis* from the common ancestor of *T. papuae* and *T. zimbabwensis* (in poikilotherms; Fig. 1a,c) coincides with the Tibetan plateau uplift and climate change around 10–8 mya^40^ and the divergence between *T. papuae* and *T. zimbabwensis* (4.9–2.3 mya), possibly contemporaneously with climate change and the Plio-Pleistocene extinction of some crocodylomorph reptiles^41^. An avian host might explain the occurrence of *T. pseudospiralis* south of the Tibetan plateau.

Radiation of encapsulated *Trichinella* involves Eurasia, Africa, North America and South America. The occurrence of *T. nelsoni* (7.8–4.1 mya) and *T. britovi*+T8 (3.2–1.7 mya) on the African continent (Fig. 1c) follows temporally discrete and independent expansion events around 7.5 mya (Miocene), 4.5–4 and 3.5 mya (Pliocene) and 2.0 mya (Pleistocene)^42^. A separation of *T. nelsoni*+*T. spiralis* near the Miocene–Pliocene boundary further establishes the basis for an independent association with hominins and humans based on ecology and later domestication of primary suid hosts for the latter species^43^. We hypothesize that the diversification of *T. britovi* and T8 took place in Africa (Fig. 1c) as a consequence of biogeographic barriers and changing environmental conditions, which is in accord with a previous suggestion by Pozio *et al.*^44^ Alternatively, the isolation and divergence of an ancestral population across Eurasia and Africa, leading to *T. britovi* and T8, with later secondary expansion, would account for the extensive geographic range of the former species^10^,^14^. We suggest that the loss of forests, formation of grasslands and the food scarcity for herbivores during the Miocene^45^,^46^ across Eurasia and Africa resulted in a massive expansion of an entirely new guild of predators/hunters that were able to follow scarce prey over vast distances in the later Miocene, Pliocene and Pleistocene^47^. Encapsulated *Trichinella* taxa initially expanded into North America across Beringia during a time frame deeper than 5 mya, when the landmass was a permanent geographic feature linking Eurasia and the Nearctic, and before the inception of Northern Hemisphere glaciations^48^. The distribution of *T. patagoniensis* is consistent with the initial emergence of the Panamanian Isthmus (∼10 mya), as recently established^49^,^50^,^51^. New chronologies are also consistent with the complex nature of faunal assembly in the Neotropical region, providing a mechanism for geographic colonization by small cats, followed by extensive radiation in South America after 8 mya, before the Great American Interchange^52^. Interestingly, there is no current evidence for geographic colonization of South America after 3 mya by nearctic species of *Trichinella* (for example, *T. murrelli* or T6), coincidental with large felids, procyonids, canids or mustelids (Fig. 1c). Inception of Northern Hemisphere glaciation cycles and periodic emergence of the Bering land bridge after 2.5 and 2.0 mya^53^ led to independent episodes of geographic colonization and host-switching, driving patterns of isolation and genetic divergence (radiation) of *Trichinella*, linking Eurasia and the Nearctic^48^. Diversification of Eurasian/Nearctic sister species, including *T. nativa+*T6, *T. murrelli*+T9 and *T. pseudospiralis* in Northern America, reflects an intricate history in response to climate variation and habitat change, facilitating independent events of biotic expansion among carnivoran and other mammalian assemblages, including ursids, canids and mustelids primarily from Eurasia into North America (Fig. 1c). In addition, we hypothesize that T9 diverged from a common ancestor with *T. murrelli* before geographic colonization of the New World, or following isolation across Bering Strait during a glacial maximum (Fig. 1c). In the absence of a fossil record for *Trichinella*, it would be useful to determine the sequence of at least one *Trichinella* taxon from an extinct, infected vertebrate (for example, carnivoran) as a reference in time to assist future studies. The chronological and spatial history of *Trichinella* is described by episodic or cyclical patterns of independent geographic and host colonization on global and regional scales, the extensive development of mosaic faunal assemblages consistent with an integrated history for taxon pulses and ecological fitting mediated by climate and habitat variation over the late Tertiary^48^,^54^,^55^.

### Parasite–host adaptation

The significantly higher GC content in the genomes and coding regions of encapsulated compared with non-encapsulated taxa (Kolmogorov–Smirnov (KS) test: *P* values=3.801 × 10^−4^ and 7.466 × 10^−4^, respectively; effect size=1) suggests that these two *Trichinella* clades may have adapted differently to varying environmental stresses (for example, temperature), host immune attacks and/or body temperatures (reptiles versus birds and mammals). We also propose that the intracellular lifestyle and associated bottleneck have resulted, throughout evolution, in a considerable reduction of genome size (49 Mb) and lower GC content (33%) in both encapsulated and non-encapsulated *Trichinella* taxa compared with extracellular relatives such as *Trichuris suis* (78.5 Mb; 44%)^22^, which accords with observations for selected pathogens^56^,^57^.

Considering the morphological differences between encapsulated and non-encapsulated *Trichinella* taxa in host muscle cells^12^, we focused on exploring ES molecules likely to be associated with parasite–host interactions or host cell modification. Although more than half (52.9%) of genes encoding putative ES proteins could not be annotated (Supplementary Data 1), we identified seven SCOs (groups (GRPs) 2,426, 4,153, 2,136, 3,720, 2,832, 3,607 and 2,897) encoding ES orphans whose gene transcription is significantly upregulated exclusively in encapsulated taxa, and which are hypothesized to modulate host immune responses or immune evasion. By contrast, we identified an SCO (GRP 2254) encoding a cathelidicin-like molecule whose transcription is upregulated only in all non-encapsulated taxa, and might suppress antigen processing and/or presentation, a proposal supported by some evidence for an orthologue (*Fh*-HDM-1) in the parasitic flatworm *Fasciola hepatica*^58^. In both encapsulated and non-encapsulated taxa, we identified an orphan protein (GRP 838; GenBank accession: BG520575) that possesses a structural motif that is consistent with mitochondrial cytochrome *c* (Supplementary Data 3) and/or the mitochondrial dynamics protein of 51 kDa (MID-51)^59^ detected previously in *T. spiralis*^30^,^60^. This ES protein likely modifies host cells by uncoupling the mitochondria to maintain an anaerobic state required by the first larval stage (L1) of *Trichinella*^61^,^62^ (Supplementary Data 3). Homologues of the 45-kDa secreted proteins^30^,^32^ (GeneBank accession: AAA20539.1) were encoded in all *Trichinella* taxa (GRP 70; Supplementary Data 3); the predicted structures of these proteins match plasminogens and thrombins (Supplementary Data 3), which are serine proteases with roles in pro-collagen production and/or the activation of collagenases^63^,^64^, respectively.

Subsequently, we identified 1,042 orthologous gene groups (GRPs) exclusively in genomes of the encapsulated taxa, and 747 were exclusive to non-encapsulated taxa (Fig. 1a). We explored the distinctiveness between all encapsulated and non-encapsulated taxa, for which RNA sequencing (RNA-seq) data were available by comparing their transcription profiles at the L1 stage for all SCOs (*n*=2,855; Supplementary Tables 3 and 4). This analysis identified SCOs that were uniquely transcribed in encapsulated (*n*=47) and non-encapsulated (*n*=68) taxa (Supplementary Data 3). Interestingly, the average GC content of all of these 115 SCOs is significantly lower (KS test: *P* value<3.059 × 10^−7^; effect size=1) than that of all genes of all *Trichinella* taxa. In addition, the significantly lower AT content of differentially transcribed genes (KS test: *P* value=4.076 × 10^−2^; effect size=0.67) in non-encapsulated taxa suggests a distinct adaptation of genes to the muscle cell compared with encapsulated taxa.

Interestingly, we found expansions in two orthologous groups in non-encapsulated taxa. The first (GRP 149) represents a serine protease precursor with two trypsin-like domains (TsSerP and AF331156)^65^, which is expanded in all non-encapsulated taxa (Supplementary Data 3) and appears to play a vital role in larval feeding and/or moulting^65^. The second group represents a multicystatin-like domain protein (MCD-1)^32^ (GRP 482) encoded by six gene copies in *T. papuae*, and two copies in *T. pseudospiralis* (T4.5) from Australia and *T. zimbabwensis* compared with one copy in all other *Trichinella* taxa. This protein contains three repeated domains, each with similarity to family 2 cystatins^66^, but lacking critical consensus sites for cysteine protease inhibition. In this respect, the domain organization is similar to that of mammalian kininogens and a known six-domain cystatin from *F. hepatica*^67^. We propose that MCD-1 might function as a cytokine antagonist by binding to transforming growth factors (TGF)-β1 and -β2 in a manner similar to fetuin^68^, a proposal that is consistent with previous findings for *T. spiralis* and evidence of low-level expression of TGF-β in the epithelium of the jejunum of infected mice^69^. Certainly, family 2 cystatins secreted by other parasitic nematodes have known roles in immune evasion, including the inhibition of proteases involved in antigen presentation and modulation of cytokine responses^70^. Although MCD-1 is unlikely to function as a typical cystatin, it is expressed at the parasite–host interface^32^ and might modulate host immune responses or enable immune evasion. An expanded set of MCD-1s might allow some non-encapsulated *Trichinella* taxa to suppress immune responses better than encapsulated taxa, possibly facilitating dissemination into a wider range of vertebrate hosts^12^.

## Discussion

Using Illumina-based sequencing and bioinformatics, we assembled draft nuclear genomes representing all 12 recognized taxa of *Trichinella*, including five geographic isolates of *T. pseudospiralis*. Using extensive amino-acid sequence data sets derived from all shared SCOs from these nuclear genomes and/or outgroup taxa, we were able to reconstruct the phylogeny of *Trichinella*. In a previous study, Zarlenga *et al.*^14^ had proposed the phylogenetic relationships of *Trichinella* species and genotypes using small DNA sequence data sets. At the time, these authors provided comprehensive interpretations of the findings and concluded that post-Miocene expansion, colonization and host-switching drove speciation among extant members of the genus *Trichinella*; although their study was very informative and interesting, the resolution of some clades and the positions of some of the taxa, such as *T. nativa* and T6 as well as *T. murrelli* and T9, did not always appear to be well supported statistically by the data presented, when outgroups (mermithids and *Trichuris* spp.) were included in the analyses. The reason for the limited resolution of some relationships was likely because of the use of sequence data set representing only selected genetic loci (nuclear small subunit and second internal transcribed spacer; mitochondrial large subunit and cytochrome *c* oxidase subunit 1)^14^. In addition, it appears that some of the species selected as outgroups might have been too distant to achieve a robust phylogeny using all of the concatenated sequence data in the analyses.

Here we utilized an extensive amino-acid sequence data set representing all SCOs (concatenated alignment over 597,495 amino-acid positions) originating from all 16 genomes representing all members of the *Trichinella* complex, including all known geographic isolates of *T. pseudospiralis* and *T. patagoniensis*, not previously available^14^,^15^, as well as *Trichuris suis* and *A. suum* (outgroups) in the analyses. By contrast, in a previous study^21^ we could not use *Trichuris* or *Ascaris* as outgroups because of excessive sequence divergence in predicted mitochondrial proteins (for example, NAD4 and NAD6) encoded by some genes, such that an unambiguous alignment of homologous characters was impossible; these outgroups therefore had to be excluded from the analyses of mitochondrial data sets, as their inclusion would have led to erroneous results and interpretation. In the present study, the definition of 1,284–2,855 SCOs among all *Trichinella* taxa (with or without outgroups) allowed, employing an iterative, stepwise approach, the reconstruction of a robust phylogeny utilizing three independent tree-building methods. In accordance with previous investigations^14^,^21^,^68^,^69^, the present analyses showed that the encapsulated taxa grouped separately from non-encapsulated taxa. Importantly, we were also able to resolve, for the first time, the phylogenetic positions of *T. spiralis*+*T. nelsoni* and *T. murrelli*+T9, five representatives of *T. pseudospiralis* and *T. patagoniensis* in relation to all other taxa, with strong (99–100%) statistical support. The diversification times of *Trichinella* matched well with known historical global events and allowed the biogeography of all taxa to be reconstructed. This biogeography clearly supports the notion that encapsulated and non-encapsulated taxa frequently occur in sympatry and in the context of faunal assembly, driven strongly by events of geographic and host colonization, involving complex spatial and chronological mosaics linking evolutionary and ecological time^54^. Nonetheless, in the future, extensive sampling of *Trichinella* taxa from around the world will be required to explore, in depth, population genetic structures to reassess the present phylogenic and biogeographic reconstruction, and to attempt to identify the host origin of ancestral forms of *T. spiralis*, the principal causative agent of human trichinellosis. In conclusion, although the present study focused sharply on fundamental phylogenetic and biogeographic aspects, the genomic and transcriptomic resources created here will substantially accelerate many fundamental and applied areas of *Trichinella*/trichinellosis research. In addition, the genome-wide approach that we employed should have applicability to a wide range of parasites and other metazoan organisms.

## Methods

### Production and procurement of *Trichinella* taxa

Sixteen samples (isolates) of first-stage larvae (L1s) representing all 12 recognized species and genotypes of *Trichinella* were produced at the International *Trichinella* Reference Center ( http://www.iss.it/site/Trichinella/), Istituto Superiore di Sanità (ISS), Rome, Italy. The 16 samples included the following: one sample of each *T. spiralis* (code: ISS3), *T. nativa* (ISS10), *T. britovi* (ISS120), *T. murrelli* (ISS417), *T. nelsoni* (ISS37), *T. patagoniensis* (ISS2496) as well as *Trichinella* genotypes T6 (ISS34), T8 (ISS272) and T9 (ISS409); five samples representing distinct geographic isolates of *T. pseudospiralis* (ISS13, ISS588, ISS176, ISS470 and ISS141=T4.1, T4.2, T4.3, T4.4 and T4.5, respectively); and one sample of each *T. papuae* (ISS1980) and *T. zimbabwensis* (ISS1029; *cf.* Tables 1 and 2). These samples were individually produced in female CD1 mice. L1s were recovered from host muscles by pepsin (1%)–HCl (1%) digestion at 40 °C for 30 min, sedimented, washed extensively in physiological saline and suspended in 90% ethanol for storage^71^. Each sample, which represented a packed volume of 50 μl of L1s, was snap-frozen in liquid nitrogen and kept at −80 °C until nucleic acid isolation.

### RNA-seq and transcriptome assembly

Total RNA was isolated separately from L1s of each isolate of *Trichinella* employing the TriPure isolation reagent (Roche Molecular Biochemicals). Packed volumes of 20–50 μl were used, equating to thousands of larvae. RNA yields were estimated spectrophotometrically (NanoDrop 1000), and the integrity of RNA was verified using the BioAnalyzer. RNA-seq was conducted using an established method^72^ and relevant data summarized (*cf*. Supplementary Tables 3 and 4). *De novo* assemblies were performed using a software pipeline, incorporating the programme Trimmomatic v.0.27 ( http://www.usadellab.org/cms/?page=trimmomatic) for read quality-filtering, Khmer v.1.1 (ref. 73) for the reduction of high and low coverage reads, and Velvet v.1.2.07 (ref. 74) and Oases v.0.2.08 (ref. 75) for sequence assembly.

### Other methods

Genome sequencing and assembly, prediction of repetitive elements, prediction of protein-encoding genes, functional annotation, phylogenetic and divergence time analyses, synteny, GC content and differential transcription analyses are described in Supplementary Methods.

## Additional information

**Accession codes:** This Whole Genome Shotgun project has been deposited in the NCBI BioProject database with accession code PRJNA257433. The project includes: all the genome assemblies under accession codes JYDH00000000 to JYDW00000000; raw Illumina read sets for the genomic DNAs; raw reads for RNA-seq under accession codes SRS672137, SRS898754, SRS900949, SRS906989, SRS906991 to SRS906994, SRS906996, SRS906998 to SRS907002, SRS908054 and SRS908055; and assembled transcriptomes for the 12 *Trichinella* taxa under accession codes GEBM00000000 to GEBP00000000, GEBR00000000 to GEBZ00000000, GECA00000000 and GECJ00000000. The versions described in this paper are JYDH01000000 to JYDW01000000 for the genomes, and GEBM01000000 to GEBP01000000, GEBR01000000 to GEBZ01000000, GECA01000000 and GECJ01000000 for the transcriptomes.

**How to cite this article:** Korhonen, P. K. *et al.* Phylogenomic and biogeographic reconstruction of the *Trichinella* complex. *Nat. Commun.* 7:10513 doi: 10.1038/ncomms10513 (2016).

## Supplementary Material

Supplementary Information Supplementary Tables 1-8, Supplementary Methods and Supplementary References

Supplementary Data 1 Predicted excretory/secretory (ES) proteins of *Trichinella* taxa encoded by single copy orthologs (SCO).

Supplementary Data 2 Syntenic correlation matrix and dendrogram for *Trichinella* taxa.

Supplementary Data 3 Single copy orthologs (SCO) that are differentially transcribed between encapsulated and non-encapsulated clades of *Trichinella*.

## Figures and Tables

**Table 1:** Assembly and gene prediction statistics for the draft genomes of all recognized encapsulated *Trichinella* taxa*.

**Description**	**T1 ISS3**	**T2 ISS10**	**T3 ISS120**	**T5 ISS417**	**T6 ISS34**	**T7 ISS37**	**T8 ISS272**	**T9 ISS409**	**T12 ISS2496**
Country of origin	Poland	Norway	Italy	USA	USA	Tanzania	Namibia	Japan	Argentina
Host of origin	Domestic pig	Polar bear	Red fox	Coyote	Grizzly bear	Warthog	Lion	Raccoon dog	Cougar
Genome size (bp)	50,035,721	48,088,508	51,516,808	49,039,267	50,855,377	47,453,436	49,332,017	49,096,069	49,773,209
Number of scaffolds; contigs	7,667; 9,209	4,635; 5,353	8,025; 9,709	5,255; 6,430	5,957; 7,471	2,973; 4,254	4,125; 5,876	5,824; 7,488	6,623; 8,003
N50 (bp)	212,546	141,195	147,150	106,482	158,103	293,867	239,129	212,690	154,103
N90 (bp)	36,812	30,368	9,232	15,500	17,717	70,070	46,127	43,251	23,618
Genome GC content (%)	33.62	33.56	33.72	33.58	33.44	33.49	33.57	33.48	33.62
Coding GC content (%)	43.08	43.14	43.31	43.14	43	43.1	43.15	43.06	43.24
Exonic proportion; including introns (%)	33.08; 71.63	33.71; 71.36	34.09; 71.23	33.62; 69.28	33.54; 69.81	33.87; 70.85	33.31; 69.28	35.26; 74.88	35.88; 72.27
Number of putative coding genes	14,745	13,662	16,067	14,863	15,242	13,232	14,920	13,127	15,319
Mean gene size (bp)	2,526	2,604	2,381	2,370	2,420	2,633	2,377	2,947	2,449
Mean CDS length (bp)	1,045	1,093	962	1,017	1,003	1,108	1,026	1,061	965
Mean exon count per gene	6.31	6.39	5.7	6.01	5.93	6.48	6.01	6.51	5.91
Mean exon length (bp)	186.2	193.9	202.09	193.32	198.14	196.76	192.52	216.2	209.06
Mean intron length (bp)	254.67	253.47	261.63	241.76	253.31	248.69	244.35	279.96	247.89
Total length of coding sequences	27,499,002	25,039,143	29,213,671	25,397,919	26,529,424	25,001,000	25,015,560	29,155,076	25,830,832
Repetitive sequences (%)	18.97	17.79	21.8	18.78	20.29	17.26	18.68	6.74	19.64
CEG completeness: complete; partial (%)	95.97; 97.18	95.97; 97.18	96.77; 97.18	95.97; 97.18	96.37; 97.18	96.37; 97.18	96.77; 97.18	95.56; 97.18	96.37; 97.18

CDS, coding DNA sequence; CEG, core essential gene; ISS, Istituto Superiore di Sanità.

International *Trichinella* Reference Center ( http://www.iss.it/site/Trichinella/) ISS codes are indicated.

^*^T1=*T. spiralis*; T2=*T. nativa*; T3=*T. britovi*; T5=*T. murrelli*; T7=*T. nelsoni*; T12=*T. patagoniensis*; and *Trichinella* genotypes T6, T8 and T9.

**Table 2:** Assembly and gene prediction statistics for the draft genomes of all recognized non-encapsulated *Trichinella* taxa*.

**Description**	**T4.1 ISS13**	**T4.2 ISS588**	**T4.3 ISS176**	**T4.4 ISS470**	**T4.5 ISS141**	**T10 ISS1980**	**T11 ISS1029**
Country of origin	Russia	Russia	Kazakhstan	USA	Australia	Thailand	Zimbabwe
Host of origin	Raccoon	Brown rat	Tawny eagle	Black vulture	Spotted quoll	Human	Nile crocodile
Genome size (bp)	49,202,366	48,147,010	49,171,591	48,479,966	46,056,875	46,871,975	50,937,231
Number of scaffolds; contigs	7,287; 8,136	7,547; 7,647	6,600; 7,483	6,287; 7,079	1,381; 2,571	2,552; 3,122	11,275; 12,675
N50 (bp)	235,426	112,255	287,133	234,172	167,180	222,396	205,645
N90 (bp)	60,266	9,250	69,797	50,288	50,779	64,723	8,776
Genome GC content (%)	32.61	32.58	32.57	32.69	32.46	32.7	32.87
Coding GC content (%)	42.64	42.39	42.44	42.62	42.24	42.25	42.41
Exonic proportion; including introns (%)	33.63; 72.68	34.73; 71.61	34.24; 73.39	29.84; 61.96	33.73; 67.98	35.96; 76.49	33.92; 72.45
Number of putative coding genes	12,699	13,754	12,462	14,708	11,006	11,854	14,933
Mean gene size (bp)	2,955	2,620	3,053	2,071	2,944	3,169	2,591
Mean CDS length (bp)	1,041	1,006	1,052	994	1,122	1,133	933
Mean exon count per gene	6.58	6.22	6.66	5.91	6.64	6.92	5.87
Mean exon length (bp)	210.71	207.52	217.94	169.78	222.84	217.53	209.41
Mean intron length (bp)	281.73	255.09	283.56	217.87	260.03	281.51	280.34
Total length of coding sequences	25,932,768	21,637,764	25,191,644	15,904,360	22,611,694	24,407,202	26,665,876
Repetitive sequences (%)	18.01	18.41	17.73	17.71	16.12	14.47	20.99
CEG completeness: complete; partial (%)	96.77; 97.58	96.37; 97.58	96.77; 97.58	97.18; 97.58	95.97; 97.58	96.37; 97.58	97.18; 97.58

CDS, coding DNA sequence; CEG, core essential gene; ISS, Istituto Superiore di Sanità.

International *Trichinella* Reference Center ( http://www.iss.it/site/Trichinella/) ISS codes are indicated.

^*^T4=*T. pseudospiralis* (including five distinct populations: T4.1–T4.5); T10=*T. papuae*; T11=*T. zimbabwensis*.
