# Supplementary material for: Phylogenomic and biogeographic reconstruction of the Trichinella complex
Source: Nat Commun. 2016 Feb 1;7:10513. doi: 10.1038/ncomms10513 (PMC4740406; doi:10.1038/ncomms10513)
Supplement: Supplementary Information — Supplementary Tables 1-8, Supplementary Methods and Supplementary References [file ncomms10513-s1.pdf]

Supplementary Table 1 | Read statistics for sequenced genomic libraries representing all recognized encapsulated *Trichinella* taxa[a]

Description	T1 ISS3	T2 ISS10	T3 ISS120	T5 ISS417	T6 ISS34	T7 ISS37	T8 ISS272	T9 ISS409	T12 ISS2496
Raw read pair count, insert size (in bp): 180	316,591,684	296,810,444	235,782,232	187,890,288	216,988,832	224,746,404	187,112,284	242,852,752	194,449,732
Raw read pair count, insert size: 250									
Raw read pair count, insert size: 300	67,751,496	158,925,252	126,298,976	111,179,232	118,779,276		113,860,560		111,039,388
Raw read pair count, insert size: 350						128,608,520		102,563,584	
Raw read pair count, insert size: 400									
Raw mate pair count, insert size: 3000 (TrueSeq)		51,956,044	76,300,796	63,071,684	85,927,768				
Raw mate pair count, insert size: 3000 (Nextera)	51,490,980					97,609,28	76,646,496	73,779,524	67,540,312
Filtered read pair count, insert size:180	299,357,540	262,007,596	215,037,496	165,906,508	195,119,440	202,955,308	169,326,852	216,246,080	175,986,276
Filtered read pair count, insert size: 250									
Filtered read pair count, insert size: 300	63,856,684	142,495,408	120,323,628	100,849,636	98,334,296		104,132,924		99,511,744
Filtered read pair count, insert size: 350						116,117,216		91,430,924	
Filtered read pair count, insert size: 400									
Filtered mate pair count, insert size: 3000 (TrueSeq)		45,989,712	64,160,916	56,431,752	72,502,280				
Filtered mate pair count, insert size: 3000 (Nextera)	48,859,732					84,746,668	66,170,532	65,753,908	60,049,520

[a]T1 = *T. spiralis*; T2 = *T. nativa*; T3 = *T. britovi*; T5 = *T. murrelli*; T7 = *T. nelsoni*; T12 = *T. patagoniensis*; and *Trichinella* genotypes T6, T8 and T9.
International *Trichinella* Reference Center (http://www.iss.it/site/Trichinella/) Istituto Superiore di Sanità (ISS) codes are indicated.

Supplementary Table 2 | Read statistics for sequenced genomic libraries representing all recognized non-encapsulated *Trichinella* taxa[a]

Description	T4.1 ISS13	T4.2 ISS588	T4.3 ISS176	T4.4 ISS470	T4.5 ISS141	T10 ISS1980	T11 ISS1029
Raw read pair count, insert size (in bp): 180							247,846,884
Raw read pair count, insert size: 250	121,370,840	177,251,764	138,448,744	108,635,224	37,645,482	203,142,876	
Raw read pair count, insert size: 300							112,401,080
Raw read pair count, insert size: 350							
Raw read pair count, insert size: 400	42,385,200	50,758,072	40,933,668	44,349,548	44,458,932	40,279,436	
Raw mate pair count, insert size: 5000 (TrueSeq)					118,393,268		
Raw mate pair count, insert size: 3000 (Nextera)	27,082,060	32,085,252	32,741,688	25,372,988		29,256,416	98,284,656
Filtered read pair count, insert size: 180							227,621,512
Filtered read pair count, insert size: 250	112,881,468	165,311,552	133,830,124	104,087,832	12,684,296	187,260,004	
Filtered read pair count, insert size: 300							101,248,916
Filtered read pair count, insert size: 350							
Filtered read pair count, insert size: 400	40,568,468	45,062,448	38,689,428	39,929,036	14,333,244	36,328,776	
Filtered mate pair count, insert size: 5000 (TrueSeq)					13,697,548		
Filtered mate pair count, insert size 3000 (Nextera)	26,373,076	30,980,268	31,529,472	24,580,236		28,178,004	86,954,640

[a]T4 = *T. pseudospiralis* (including five distinct populations: T4.1 to T4.5); T10 = *T. papuae*; T11 = *T. zimbabwensis*.
International *Trichinella* Reference Center (http://www.iss.it/site/Trichinella/) Istituto Superiore di Sanità (ISS) codes are indicated.

Supplementary Table 3 | Read statistics for sequenced RNA-seq libraries representing all recognized encapsulated *Trichinella* taxa[a]

Description	T1 ISS3	T2 ISS10	T3 ISS120	T5 ISS417	T6 ISS34	T7 ISS37	T8 ISS272	T9 ISS409	T12 ISS2496
Country of origin	Poland	Norway	Italy	USA	USA	Tanzania	Namibia	Japan	Argentina
Host of origin	Domestic pig	Polar bear	Red fox	Coyote	Grizzly bear	Warthog	Lion	Raccoon dog	Cougar
Raw read pair count	14,929,613	5,317,461	6,611,322	6,378,894	7,010,637	5,679,900	5,984,891	13,123,322	14,038,915
Filtered read pair count	14,004,989	4,708,644	5,872,592	5,600,848	6,229,405	5,013,318	5,285,008	11,133,664	2,484,171

[a]T1 = *T. spiralis*; T2 = *T. nativa*; T3 = *T. britovi*; T5 = *T. murrelli*; T7 = *T. nelsoni*; T12 = *T. patagoniensis*; and *Trichinella* genotypes T6, T8 and T9.
International *Trichinella* Reference Center (http://www.iss.it/site/Trichinella/) Istituto Superiore di Sanità (ISS) codes are indicated.

Supplementary Table 4 | Read statistics for RNA-seq libraries representing all recognized non-encapsulated *Trichinella* taxa[a]

Description	T4.1 ISS13	T4.2 ISS588	T4.3 ISS176	T4.4 ISS470	T4.5 ISS141	T10 ISS1980	T11 ISS1029
Country of origin	Russia	Russia	Kazakhstan	USA	Australia	Thailand	Zimbabwe
Host of origin	Raccoon	Brown rat	Tawny eagle	Black vulture	Spotted quoll	Human	Nile crocodile
Raw read pair count	12,189,135	11,182,791	14,426,115	NA	12,799,251	10,574,527	11,106,742
Filtered read pair count	10,161,999	9,076,764	12,103,972	NA	2,646,364	9,114,194	9,300,102

[a]T4 = *T. pseudospiralis* (including five distinct populations: T4.1 to T4.5); T10 = *T. papuae*; T11 = *T. zimbabwensis*.
International *Trichinella* Reference Center (http://www.iss.it/site/Trichinella/) Istituto Superiore di Sanità (ISS) codes are indicated.

Supplementary Table 5 | Statistics of repeats in the genomes of all recognized encapsulated *Trichinella* taxa[a]

Description	T1 ISS3	T2 ISS10	T3 ISS120	T5 ISS417	T6 ISS34	T7 ISS37	T8 ISS272	T9 ISS409	T12 ISS2496
Coverage (bp; %)	9,492,547; 19.0	8,555,586; 17.8	11,230,493; 21.8	9,209,516; 18.8	10,316,475; 20.3	8,192,708; 17.3	9,217,257; 18.7	3,311,482; 6.7	9,774,581; 19.6
DNA transposons (bp; %)	1,725,722; 3.4	2,163,297; 4.5	2,832,995; 5.5	1,681,718; 3.4	2,197,565; 4.3	2,030,012; 4.3	1,673,874; 3.4	341,470; 0.7	1,500,649; 3.0
Retrotransposons (bp; %)	1,111,727; 2.2	1,849,709; 3.8	1,901,051; 3.7	1,358,182; 2.8	1,342,882; 2.6	1,043,636; 2.2	1,320,874; 2.7	561,105; 1.1	1,645,626; 3.3
Unclassified interspersed (bp; %)	4,555,218; 9.1	2,285,271; 4.8	4,236,724; 8.2	4,096,047; 8.4	4,596,149; 9.0	2,994,969; 6.3	4,084,061; 8.3	17,269; 0.0	4,478,206; 9.0
Total interspersed (bp; %)	7,392,667; 14.8	6,298,277; 13.1	8,970,770; 17.4	7,135,947; 14.6	8,136,596; 16.0	6,068,617; 12.8	7,078,809; 14.3	919,844; 1.9	7,624,481; 15.3
Simple repeats (bp; %)	2,075,522; 4.1	2,222,321; 4.6	2,232,971; 4.3	2,041,001; 4.2	2,153,209; 4.2	2,092,563; 4.4	2,114,326; 4.3	2,360,121; 4.8	2,106,638; 4.2

[a]T1 = *T. spiralis*; T2 = *T. nativa*; T3 = *T. britovi*; T5 = *T. murrelli*; T7 = *T. nelsoni*; T12 = *T. patagoniensis*; and *Trichinella* genotypes T6, T8 and T9. International *Trichinella* Reference Center (http://www.iss.it/site/Trichinella/) Istituto Superiore di Sanità (ISS) codes are indicated.

Supplementary Table 6 | Statistics of repeats in the genomes representing all recognized non-encapsulated *Trichinella* taxa[a]

Description	T4.1 ISS13	T4.2 ISS588	T4.3 ISS176	T4.4 ISS470	T4.5 ISS141	T10 ISS1980	T11 ISS1029
Coverage (bp; %)	8,859,250; 18.0	8,861,862; 18.4	8,719,098; 17.7	8,585,342; 17.7	7,426,141; 16.1	6,782,587; 14.5	10,689,225; 21.0
DNA transposons (bp; %)	599,152; 1.2	938,332; 1.9	629,768; 1.3	566,165; 1.2	1,284,221; 2.8	1,341,232; 2.9	1,031,858; 2.0
Retrotransposons (bp; %)	845,982; 1.7	818,101; 1.7	632,756; 1.3	1,094,191; 2.3	846,565; 1.8	760,502; 1.6	1,259,551; 2.5
Unclassified interspersed (bp; %)	4,763,549; 9.7	4,427,993; 9.2	4,789,231; 9.7	4,123,993; 8.5	3,183,746; 6.9	2,095,504; 4.5	5,396,847; 10.6
Total interspersed (bp; %)	6,208,683; 12.6	6,184,426; 12.8	6,051,755; 12.3	5,784,349; 11.9	5,314,532; 11.5	4,197,238; 9.0	7,688,256; 15.1
Simple repeats (bp; %)	2,621,310; 5.3	2,633,991; 5.5	2,649,228; 5.4	2,766,410; 5.7	2,091,270; 4.5	2,555,139; 5.5	2,968,049; 5.8

[a]T4 = *T. pseudospiralis* (including five distinct populations: T4.1 to T4.5); T10 = *T. papuae*; T11 = *T. zimbabwensis*.
International *Trichinella* Reference Center (http://www.iss.it/site/Trichinella/) Istituto Superiore di Sanità (ISS) codes are indicated.

Supplementary Table 7 | Annotation statistics of proteins to be encoded in the genomes of all recognized encapsulated *Trichinella* taxa[a]

Description<br>No. of matches listed for:	T1 ISS3	T2 ISS10	T3 ISS120	T5 ISS417	T6 ISS34	T7 ISS37	T8 ISS272	T9 ISS409	T12 ISS2496
InterPro	5,899	6,011	6,372	6,054	6,130	5,894	6,242	5,620	6,137
InterPro PIRSF	246	251	246	248	253	254	263	234	256
InterPro PRINTS	1,030	1,005	986	984	998	1,005	1,022	929	1,021
InterPro PANTHER	5,566	5,613	5,879	5,655	5,700	5,564	5,827	5,261	5,751
InterPro Pfam	5,088	5,220	5,362	5,165	5,305	5,085	5,312	4,835	5,176
Homologous proteins	10,858	10,063	11,931	10,940	11,347	9,932	11,123	9,927	11,283
Caenorhabditis elegans homologs	5,314	5,331	5,300	5,254	5,347	5,232	5,365	4,960	5,280
KEGG	10,891	10,062	12,000	10,989	11,394	9,979	11,167	9,968	11,304
NCBI nr	10,855	10,060	11,930	10,939	11,345	9,927	11,121	9,924	11,277
SwissProt	5,653	5,775	5,912	5,726	5,881	5,662	5,865	5,389	5,838
TMHMM	1,604	1,485	1,564	1,549	1,553	1,550	1,606	1,402	1,583
Phobius and SignalP	772	786	820	815	847	777	843	737	795
Predicted secretome	357	343	381	386	387	363	400	314	407

[a]T1 = *T. spiralis*; T2 = *T. nativa*; T3 = *T. britovi*; T5 = *T. murrelli*; T7 = *T. nelsoni*; T12 = *T. patagoniensis*; and *Trichinella* genotypes T6, T8 and T9.
International *Trichinella* Reference Center (http://www.iss.it/site/Trichinella/) Istituto Superiore di Sanità (ISS) codes are indicated.

Supplementary Table 8 | Annotation statistics of proteins predicted to be encoded in the genomes of all recognized non-encapsulated *Trichinella* taxa[a]

Description<br>No. of matches listed for:	T4.1 ISS13	T4.2 ISS588	T4.3 ISS176	T4.4 ISS470	T4.5 ISS141	T10 ISS1980	T11 ISS1029
InterPro	5,388	5,585	5,289	5,890	5,756	5,607	5,672
InterPro PIRSF	248	261	241	266	254	255	260
InterPro PRINTS	958	989	950	1,037	986	991	973
InterPro PANTHER	5,106	5,291	5,018	5,569	5,454	5,273	5,294
InterPro Pfam	4,692	4,869	4,626	5,128	4,951	4,870	4,869
Homologous proteins	7,856	8,204	7,659	8,768	8,174	8,229	8,859
Caenorhabditis elegans homologs	4,973	5,128	4,891	5,415	5,396	5,047	5,046
KEGG	7,893	8,234	7,691	8,814	8,192	8,220	8,875
NCBI nr	7,854	8,202	7,655	8,767	8,171	8,222	8,848
SwissProt	5,208	5,390	5,143	5,662	5,625	5,444	5,482
TMHMM	1,409	1,509	1,380	1,711	1,313	1,435	1,557
Phobius and SignalP	723	770	713	878	742	747	782
Predicted secretome	314	355	316	414	334	346	384

[a]T4 = *T. pseudospiralis* (including five distinct populations: T4.1 to T4.5); T10 = *T. papuae*; T11 = *T. zimbabwensis*.
International *Trichinella* Reference Center (http://www.iss.it/site/Trichinella/) Istituto Superiore di Sanità (ISS) codes are indicated.

Supplementary Methods

Genomic sequencing and assembly. High molecular weight genomic DNAs were isolated from L1s of individual *Trichinella* taxa using an established protocol[1]. DNA amounts were estimated using a *Qubit* fluorometer dsDNA *HS Kit (Invitrogen), according to the manufacturer's instructions. Genomic* DNA integrity was verified by agarose gel electrophoresis and using a BioAnalyzer (2100, Agilent). Paired-end (180 bp and 300 bp inserts) and mate pair (3,000 bp insert) genomic libraries were constructed and assessed for both size distribution and quality using the BioAnalyzer. Genomic sequencing was conducted using HiSeq or MiSeq Illumina sequencers (2 x 101 bp or 2 x 211 bp reads) employing TrueSeq or Nextera library construction protocols (Illumina). For all libraries, reads were exported to FASTQ format[2], and statistics assessed (cf. Supplementary Tables 1 and 2). The shotgun assembly and scaffolding from short-read data were performed using a dedicated assembly pipeline utilising the Trimmomatic quality filter[3], BayesHammer read error corrector[4], SPAdes v.2.5 assembler[5], Opera v.1.3.1 scaffolder[6] and GapFiller gap closer[7]. Both Opera and GapFiller were run three times (iteratively) or until the number of the concatenated contigs/scaffolds no longer improved.

Prediction of repetitive elements. First, genomic repeats were modelled utilising the program RepeatModeler (http://www.repeatmasker.org), which merged repeat predictions using programs RECON[8] and RepeatScout[9]. Then, modelled repeats were combined with a collection of known repeats in Repbase v.17.02[10] and soft-masked using RepeatMasker v.open-3.3.0 (http://www.repeatmasker.org).

Prediction of protein-encoding genes. Both *ab initio* and evidence-based gene predictions were conducted, and a consensus prediction was made. The programs AUGUSTUS[11], GeneMark[12, 13] and SNAP[14] were used for *ab initio* gene predictions. AUGUSTUS and SNAP were trained by combining CEGs, inferred using the program CEGMA[15], with mRNAs predicted *de novo* from assembled transcriptomes and homologous nucleotide sequences from the NCBI nt database. First, GeneMark gene predictions were used to acquire homologous cDNAs and mRNAs from NBCI nt database[16]. Second, all *de novo*-assembled transcripts for *Trichinella* taxa were run against the genome assembly using the program BLAT[17] and then filtered for full-length open reading frames (ORFs), ensuring the validity of splice sites. The nucleotide sequences and ORFs extracted were then combined with the CEG set. For each genome, the resultant, combined nucleotide sequence set was then used to train the *ab initio* gene prediction programs AUGUSTUS and SNAP to produce respective Hidden Markov models (HMM). RNA-seq reads representing the transcriptomes of individual *Trichinella* taxa were then processes using the programs TopHat[18] and Cufflinks[19] to infer genes and exon-intron boundaries in genomic scaffolds. The proteomes of each *Caenorhabditis elegans*[20] and *Trichuris suis* (male and female)[21], the predicted genes with exon-intron boundary estimates, HMMs and the combined nucleotide sequence set were then subjected to analysis using MAKER2[22], in order to provide a consensus set of predicted genes in the genome of each *Trichinella* taxon. Genes inferred to encode peptides of ≥ 30 amino acids in length were preserved. All protein-encoding gene sequences were compared by BLAST+[23, 24] (*E*-value: ≤ 10^{-8}) against those of bacteria, viruses, fungi and mouse, all available in the NCBI nt database[25], and extraneous (i.e. putatively contaminating) sequences removed. Contigs of ≤ 500 bp in length containing only one putative contaminant (*E*-value: ≤ 10^{-15}), or a prediction of an *ab initio* single exon open reading frame (ORF), were eliminated. In addition, contigs of ≤ 500 bp in length containing multiple putative contaminants (*E*-value: ≤ 10^{-8}), and/or predictions of multiple *ab initio* single exon ORFs were also removed.

Functional annotation. Genes were annotated using the programs InterProScan[26, 27] and BLAST+[23, 24]. Databases encompassed in InterProScan were PANTHER[28], PIRSF[29] and PRINTS[30]. The BLAST databases employed were NCBI protein nr[16], UniProtKB/Swiss-Prot[31] and KEGG[32, 33]. KEGG Orthology (KO) terms were inferred from KEGG BLAST (*E*-value: <

10^{-5}), with no more than five unassigned matches of less than this *E*-value[34]. An unassigned match was defined as a KEGG BLAST match without an assigned KO term in the description line. For each gene, KO terms identified were then assigned to KEGG pathways by mapping KO terms to KEGG Orthology Based Annotation System (KOBAS) database[35]. Signal peptides were predicted using the programs Phobius[36] and SignalP[37]; no KDEL retention signal motif[38] was permitted for predicted signal peptides. Individual secretomes were predicted using the program MultiLoc2[39]. A custom script together with Genome Annotation Generator (GAG) software v.1.0 (http://genomeannotation.github.io/GAG) was used to convert the assembly, the predicted genes and the gene annotations into an Abstract Syntax Notation One (ASN.1) - formatted file for NCBI submission.

Phylogenetic and divergence time analyses. As a first step, SCOs between or among the genomes representing *Trichinella* taxa and/or the outgroups *Trichuris suis* (Enoplida) and *Ascaris suum* (Ascaridida)[21, 40] were identified. SCOs were defined based on orthologous protein clusters using the program OrthoMCL[41] with NCBI-BLAST[23]. Protein sequences encoded by SCOs were extracted from resultant clusters. For these SCOs, individual amino acid sequences were aligned (selecting a minimum gap-free alignment length of 20 amino acids, with at least one phylogenetically informative site, at which at least one amino acid was distinct from all others in the alignment) using the programs MAFFT[42] and GUIDANCE[43], concatenated and then subjected to phylogenetic analyses using the methods Bayesian inference (BI) in MrBayes v.3.2.2[44, 45], maximum likelihood (ML) in RAxML v.8.0.24[46] and maximum parsimony (MP) in PAUP* v.4.0 beta (http://paup.csit.fsu.edu/index.html), using *Trichuris suis* and *Ascaris suum* as outgroups, and *T. spiralis* (ISS195)[47] for comparison. Specifically, for BI analysis, the prior evolution model for amino acids was set to JTT[48] and the likelihood model was set to invgamma[49, 50] following the model evaluation using program ProtTest 3.4[51]; the number of Markov Chain Monte Carlo (MCMC)[52, 53, 54] iterations was 100,000, from which the first 25,000 were discarded as non-converged burn-in; nodal support values were given as posterior probabilities. For ML, the same concatenated alignments were subjected to analysis using the JTT evolution model; concatenated alignment blocks were bootstrapped 100 times in RAxML to infer nodal support values. For MP, an heuristic search, utilising tree bisection and reconnection (TBR), was employed for concatenated alignments. The concatenated amino acid sequence block was bootstrapped 1,000 times using PAUP*, and estimated branch lengths included in the resultant trees. The resultant, bootstrapped trees were then subjected to analysis in the program SumTrees in the DendroPy v.3.12.0[55] python library to produce a consensus tree and to infer the nodal support values. Trees were drawn using the program FigTree v.1.4 (http://tree.bio.ed.ac.uk/software/figtree).

The difference in rate of evolution, coefficient of variation (CoV)[56], was estimated from aligned SCOs (first and second codon positions) using the program BEAST2[57]. SCOs were sorted based on their CoV, selected from the linear part of the curve and concatenated. Then, the nucleotide data were divided into three parts, according to individual codon positions, and subjected to analysis using program MCMCTREE in PAML v4.8 suite[58] employing the nucleotide substitution model HKY85+GAMMA with an uncorrelated relaxed clock model and skew normal (SN) fossil calibration distribution using the following parameters: location = 415, scale = 60, alpha = 2)[59]. To confirm the convergence of Markov Chain to a single stationary distribution, the analysis was repeated twice with and without the most closely related outgroup (*Trichuris suis*).

Synteny. SCOs common to all 16 genomes representing all 12 recognized *Trichinella* taxa were used to define the genomic anchor regions in genomic scaffolds. First, using the program OrthoCluster[60], syntenic correlation values[61] were calculated among all *Trichinella* taxa, and hierarchical clustering (Ward method)[62] was employed to construct a dendrogram. Then, using a custom script, the syntenic relationships of genomic scaffolds between *T. spiralis* and *T. nelsoni* were established in a pairwise manner, requiring each nominated scaffold-pair to share at least 15 SCOs. Scaffold-pairs were converted to a bipartite graph and then subjected to two-sided crossing minimization[63] using R programming language v.2.15 (https://www.r-

project.org) employing the program lpSolve v.5.6.10 (http://cran.r-project.org/package=lpSolve). Resultant scaffolds were then fixed in their positions or reverse complemented if crossing could be minimized. Second, the one-sided crossing minimization algorithm[64] was employed to extend the comparison with the scaffolds of *T. nelsoni* and then *T. patagoniensis*, requiring at least 10 SCOs to be shared by each scaffold pair compared. This process was extended to the genomes of all other *Trichinella* taxa in the following order: *T. nativa*, T6, T9, *T. murrelli*, T8, *T. britovi*, *T. papuae*, *T. zimbabwensis* and *T. pseudospiralis* (i.e. T4.1, T4.2, T4.3, T4.4 and T4.5), following their genetic relatedness in the consensus phylogenetic tree (cf. Fig. 1a).

GC content. The significance of the GC content differences between encapsulated and non-encapsulated clades was estimated using the Kolmogorov-Smirnov (KS) one-directional test in the R language environment.

Differential transcription analyses. The RNA-seq based differential gene transcription analyses between encapsulated (i.e. *T. spiralis*, *T. nativa*, *T. britovi*, *T. murrelli*, *T. nelsoni*, *T. patagoniensis*, and *Trichinella* T6, T8 and T9) and non-encapsulated (i.e. five distinct geographic isolates of *T. pseudospiralis*, *T. papuae* and *T. zimbabwensis*) *Trichinella* taxa were conducted using the program edgeR[65] in the R language environment. SCOs common to all *Trichinella* taxa represented the gene set used in analyses. First, reads from each paired-end (PE) RNA-seq library were filtered for quality (Phred $\geq$ 20) using the program Trimmomatic. Second, the filtered reads were mapped to the respective predicted gene set of cDNAs using Burrows-Wheeler Aligner (BWA) software[66]; the mapped reads per cDNA were counted using SAMtools program[67] and then normalised based on gene length and using the TMM method[68]. Third, differential transcription analysis was performed between the nine encapsulated and the seven non-encapsulated taxa using the program edgeR. Genes with false discovery rate (FDR) of $\leq$ 0.0001 were identified as differentially transcribed.

Supplementary References

1. Sambrook, J., Fritsch, E.F. & Maniatis, T. *Molecular Cloning: A Laboratory Manual; Second Edition.* (Cold Spring Harbor Laboratory Press, New York, U. S. A., 1987).
2. Cock, P.J., Fields, C.J., Goto, N., Heuer, M.L. & Rice, P.M. The Sanger FASTQ file format for sequences with quality scores, and the Solexa/Illumina FASTQ variants. *Nucleic Acids Res* **38**, 1767-1771 (2010).
3. Bolger, A.M., Lohse, M. & Usadel, B. Trimmomatic: a flexible trimmer for Illumina sequence data. *Bioinformatics* **30**, 2114-20 (2014).
4. Nikolenko, S.I., Korobeynikov, A.I. & Alekseyev, M.A. BayesHammer: Bayesian clustering for error correction in single-cell sequencing. *BMC Genomics* **14** Suppl 1, S7 (2013).
5. Bankevich, A. *et al.* SPAdes: a new genome assembler and its applications to single cell sequencing. *J Comput Biol* 19, 455-477 (2012).
6. Gao, S., Sung, W.-K. & Nagarajan, N. Opera: Reconstructing optimal genomic scaffolds with high-throughput paired-end sequences. *J Comput Biol* **18**, 1681-1691 (2011).
7. Boetzer, M. & Pirovano, W. Toward almost closed genomes with GapFiller. *Genome Biol* **13**, R56 (2012).
8. Bao, Z. & Eddy, S.R. Automated *de novo* identification of repeat sequence families in sequenced genomes. *Genome Res* **12**, 1269-1276 (2002).
9. Price, A.L., Jones, N.C. & Pevzner, P.A. De novo identification of repeat families in large genomes. *Bioinformatics* **21** Suppl 1, i351-358 (2005).
10. Jurka, J. *et al.* Repbase Update, a database of eukaryotic repetitive elements. *Cytogenet Genome Res* **110**, 462-467 (2005).
11. Stanke, M. & Waack, S. Gene prediction with a hidden Markov model and a new intron submodel. *Bioinformatics* **19** Suppl 2, ii215-25 (2003).
12. Lukashin, A.V. & Borodovsky, M. GeneMark.hmm: new solutions for gene finding. *Nucleic Acids Res* **26**, 1107-15 (1998).

13. Lomsadze, A., Ter-Hovhannisyan, V., Chernoff, Y.O. & Borodovsky, M. Gene identification in novel eukaryotic genomes by self-training algorithm. *Nucleic Acids Res* **33**, 6494-6506 (2005).
14. Korf, I. Gene finding in novel genomes. *BMC Bioinformatics* **5**, 59 (2004).
15. Parra, G., Bradnam, K. & Korf, I. CEGMA: a pipeline to accurately annotate core genes in eukaryotic genomes. *Bioinformatics* **23**, 1061-7 (2007).
16. Pruitt, K.D., Tatusova, T. & Maglott, D.R. NCBI Reference Sequence (RefSeq): a curated non-redundant sequence database of genomes, transcripts and proteins. *Nucleic Acids Res* **33**, D501-504 (2005).
17. Kent, W.J. BLAT - The BLAST-like alignment tool. *Genome Research* 12, 656-664 (2002).
18. Trapnell, C., Pachter, L. & Salzberg, S.L. TopHat: discovering splice junctions with RNA-Seq. *Bioinformatics* **25**, 1105-1111 (2009).
19. Roberts, A., Pimentel, H., Trapnell, C. & Pachter, L. Identification of novel transcripts in annotated genomes using RNA-Seq. *Bioinformatics* **27**, 2325-2329 (2011).
20. Harris, T.W. *et al.* WormBase: a comprehensive resource for nematode research. *Nucleic Acids Res* **38**, D463-467 (2010).
21. Jex, A.R. *et al.* Genome and transcriptome of the porcine whipworm *Trichuris suis*. *Nat Genet* **46**, 701-706 (2014).
22. Holt, C. & Yandell, M. MAKER2: an annotation pipeline and genome-database management tool for second-generation genome projects. *BMC Bioinformatics* **12**, 491 (2011).
23. Altschul, S.F., Gish, W., Miller, W., Myers, E.W. & Lipman, D.J. Basic local alignment search tool. *J Mol Biol* **215**, 403-410 (1990).
24. Camacho, C. *et al.* BLAST+: architecture and applications. *BMC Bioinformatics* **10**, 421 (2009).
25. Pruitt, K.D., Tatusova, T., Klimke, W. & Maglott, D.R. NCBI Reference sequences: current status, policy and new initiatives. *Nucleic Acids Res* **37**, D32-36 (2009).
26. Zdobnov, E.M. & Apweiler, R. InterProScan - an integration platform for the signature-recognition methods in InterPro. *Bioinformatics* **17**, 847-848 (2001).
27. Quevillon, E. *et al.* InterProScan: protein domains identifier. *Nucleic Acids Res* **33**, W116-120 (2005).
28. Mi, H., Muruganujan, A. & Thomas, P.D. PANTHER in 2013: modeling the evolution of gene function, and other gene attributes, in the context of phylogenetic trees. *Nucleic Acids Res* **41**, D377-386 (2013).
29. Nikolskaya, A.N., Arighi, C.N., Huang, H., Barker, W.C. & Wu, C.H. PIRSF Family Classification System for Protein Functional and Evolutionary Analysis. *Evol Bioinform* **2**, 197-209 (2006).
30. Attwood, T.K. *et al.* The PRINTS database: a fine-grained protein sequence annotation and analysis resource--its status in 2012. *Database (Oxford)* **2012**, bas019 (2012).
31. Magrane, M. & Consortium, U. UniProt knowledgebase: a hub of integrated protein data. *Database (Oxford)* **2011**, bar009 (2011).
32. Kanehisa, M., Goto, S., Sato, Y., Furumichi, M. & Tanabe, M. KEGG for integration and interpretation of large-scale molecular datasets. *Nucleic Acids Res* **40**, D109-114 (2012).
33. Kanehisa, M. & Goto, S. KEGG: kyoto encyclopedia of genes and genomes. *Nucleic Acids Res* **28**, 27-30 (2000).
34. Mao, X.Z., Cai, T., Olyarchuk, J.G. & Wei, L.P. Automated genome annotation and pathway identification using the KEGG Orthology (KO) as a controlled vocabulary. *Bioinformatics* **21**, 3787-3793 (2005).
35. Xie, C. *et al.* KOBAS 2.0: a web server for annotation and identification of enriched pathways and diseases. *Nucleic Acids Res* **39**, W316-322 (2011).
36. Kall, L., Krogh, A. & Sonnhammer, E.L. A combined transmembrane topology and signal peptide prediction method. *J Mol Biol* **338**, 1027-1036 (2004).
37. Petersen, T.N., Brunak, S., von Heijne, G. & Nielsen, H. SignalP 4.0: discriminating signal peptides from transmembrane regions. *Nat Methods* **8**, 785-786 (2011).
38. Pelham, H.R. The retention signal for soluble proteins of the endoplasmic reticulum. *Trends Biochem Sci* **15**, 483-486 (1990).
39. Blum, T., Briesemeister, S. & Kohlbacher, O. MultiLoc2: integrating phylogeny and gene ontology terms improves subcellular protein localization prediction. *BMC Bioinformatics* **10**, 274 (2009).
40. Jex, A.R. *et al.* *Ascaris suum* draft genome. *Nature* **479**, 529-533 (2011).

41. Li, L., Stoeckert, C.J., Jr. & Roos, D.S. OrthoMCL: identification of ortholog groups for eukaryotic genomes. *Genome Res* **13**, 2178-2189 (2003).
42. Katoh, K., Kuma, K., Toh, H. & Miyata, T. MAFFT version 5: improvement in accuracy of multiple sequence alignment. *Nucleic Acids Res* **33**, 511-518 (2005).
43. Penn, O., Privman, E., Landan, G., Graur, D. & Pupko, T. An alignment confidence score capturing robustness to guide tree uncertainty. *Mol Biol Evol* **27**, 1759-1767 (2010).
44. Ronquist, F. & Huelsenbeck, J.P. MrBayes 3: Bayesian phylogenetic inference under mixed models. *Bioinformatics* **19**, 1572-1574 (2003).
45. Altekar, G., Dwarkadas, S., Huelsenbeck, J.P. & Ronquist, F. Parallel Metropolis coupled Markov chain Monte Carlo for Bayesian phylogenetic inference. *Bioinformatics* **20**, 407-415 (2004).
46. Stamatakis, A., Ludwig, T. & Meier, H. RAxML-III: a fast program for maximum likelihood-based inference of large phylogenetic trees. *Bioinformatics* **21**, 456-463 (2005).
47. Mitreva, M. *et al.* The draft genome of the parasitic nematode *Trichinella spiralis*. *Nat Genet* **43**, 228-235 (2011).
48. Jones, D.T., Taylor, W.R. & Thornton, J.M. The rapid generation of mutation data matrices from protein sequences. *Comput Appl Biosci* **8**, 275-282 (1992).
49. Yang, Z.H. maximum-likelihood-estimation of phylogeny from dna-sequences when substitution rates differ over sites. *Mol Biol Evol* **10**, 1396-1401 (1993).
50. Yang, Z. Maximum likelihood phylogenetic estimation from DNA sequences with variable rates over sites: approximate methods. *J Mol Evol* **39**, 306-14 (1994).
51. Darriba, D., Taboada, G.L., Doallo, R. & Posada, D. ProtTest 3: fast selection of best-fit models of protein evolution. *Bioinformatics* **27**, 1164-1165 (2011).
52. Metropolis, N., Rosenbluth, A.W., Rosenbluth, A.H., Teller, H. & Teller, E. Equations of state calculations by fast computing machines. *J Chem Phys* **21**, 1087-1091 (1953).
53. Hastings, W.K. Monte-Carlo sampling methods using Markov chains and their applications. *Biometrika* **57**, 97-109 (1970).
54. Geyer, C.J. Markov-Chain Monte-Carlo Maximum-Likelihood. *Computing Science and Statistics: Proceedings of the 23rd Symposium on the Interface. Interface Foundation, Fairfax Station*, pp. 156-163 (1991).
55. Sukumaran, J. & Holder, M.T. DendroPy: a Python library for phylogenetic computing. *Bioinformatics* **26**, 1569-1571 (2010).
56. Drummond, A.J. & Bouckaert, R.R. Bayesian Evolutionary Analysis with BEAST. Cambridge University Press, UK (2015).
57. Bouckaert, R. *et al.* BEAST 2: a software platform for Bayesian evolutionary analysis. *PLoS Comput Biol* **10**, e1003537 (2014).
58. Yang, Z. PAML 4: phylogenetic analysis by maximum likelihood. *Mol Biol Evol* **24**, 1586-1591 (2007).
59. Azzalini, A. & Genton, M.G. Robust likelihood methods based on the skew-t and related distributions. *Int Stat Rev* 76, 106-129 (2008).
60. Xinghuo, Z. *et al.* OrthoCluster: a new tool for mining synteny blocks and applications in comparative genomics. in *11th international conference on Extending database technology: Advances in database technology* 656-667 (ACM, 2008).
61. Housworth, E.A. & Postlethwait, J. Measures of synteny conservation between species pairs. *Genetics* **162**, 441-448 (2002).
62. Ward, J.H. Hierarchical grouping to optimize an objective function. *J Am Stat Assoc* **58**, 236-244 (1963).
63. Zheng, L.B. & Buchheim, C. A new exact algorithm for the two-sided crossing minimization problem, in *Combinatorial Optimization and Applications, First International Conference, COCOA* (A. W. M. Dress, Y. Xu, and B. Zhu, eds.), vol. **4616** of *LNCS*, Springer, pp. 301-310 (2007).
64. Jünger, M. & Mutzel, P. 2-layer straightline crossing minimization: performance of exact and heuristic algorithms. *J Graph Algorithms Appl* **1**, 1-25.
65. Robinson, M.D., McCarthy, D.J. & Smyth, G.K. edgeR: a Bioconductor package for differential expression analysis of digital gene expression data. *Bioinformatics* **26**, 139-140 (2010).
66. Li, H. & Durbin, R. Fast and accurate long-read alignment with Burrows-Wheeler transform. *Bioinformatics* **26**, 589-595 (2010).
67. Li, H. *et al.* The Sequence Alignment/Map format and SAMtools. *Bioinformatics* **25**, 2078-2079 (2009).

68. Robinson, M.D. & Oshlack, A. A scaling normalization method for differential expression analysis of RNA-seq data. *Genome Biol* **11**, R25 (2010).
